# Epidemiology of pertussis in Saudi Arabia (2006–2023): regional variations and COVID-19 effects

**DOI:** 10.3389/fpubh.2025.1726013

**Published:** 2026-01-12

**Authors:** Ibrahim G. Alghamdi

**Affiliations:** Public Health Department, College of Applied Medical Sciences, University of Al-Baha, Al-Baha, Saudi Arabia

**Keywords:** pertussis, Saudi Arabia, whooping cough, epidemiology, incidence, COVID-19, regional variation

## Abstract

**Background:**

Pertussis remains a public health concern. This study investigated the epidemiology of pertussis in Saudi Arabia from 2006 to 2023 by examining trends in case numbers and crude incidence rates (CIR) by year, age group, region, sex, and nationality. Particular attention was given to the rise in cases during the COVID-19 pandemic (2019–2020) and its resurgence in 2023.

**Methods:**

A retrospective analysis was conducted using pertussis data reported across 20 regions of Saudi Arabia between January 2006 and December 2023 obtained from the Statistical Yearbooks of the Saudi Ministry of Health. Statistical analyses were performed using SPSS version 20.0, and both parametric and nonparametric methods were applied.

**Results:**

The incidence of pertussis showed marked fluctuations, with a sharp increase in 2019 (CIR: 0.75/100,000), a decline in 2020–2022, and a rebound in 2023 (CIR: 0.30/100,000). The highest CIRs were recorded for Qassim (0.36), Aseer (0.29), Taif (0.28), and Al-Ahsa (0.25). Regions such as Al-Baha, Jouf, and Qurayyat have reported negligible or no cases. Children aged 0–4 years accounted for the highest incidence (3.4/100,000), whereas older age groups had substantially lower rates. The male-to-female ratios were nearly equal (1.1:1 for Saudis and 1:1 for non-Saudis). Overall, the CIR in Saudi nationals was 3.6 times higher than that in non-Saudis.

**Conclusion:**

The incidence of pertussis in Saudi Arabia varies by age and region, with the greatest burden observed in young children and in regions such as Qassim and Aseer. These findings underscore the importance of targeted vaccination strategies and sustained public health efforts to address these disparities and protect vulnerable populations.

## 1 Introduction

Pertussis, commonly known as *whooping cough* or the “cough of 100 days,” is a highly contagious respiratory infection caused by *Bordetella pertussis*. The disease was first described during the Paris epidemic in 1578 and the causative organism was identified in 1906. The introduction of pertussis vaccines in the 1940s dramatically reduced the burden, especially among infants, who were once a leading cause of morbidity and mortality. Despite widespread vaccination, pertussis remains a global public health concern owing to waning immunity and the periodic resurgence of cases. Recent studies highlight factors such as reduced vaccine effectiveness and pathogen evolution as major contributors to these outbreaks (1–4).

Before vaccination became available, pertussis imposed a substantial global burden, causing over 300 million childhood deaths annually (5). The launch of the World Health Organization’s Expanded Program on Immunization (EPI) markedly reduced global morbidity and mortality rates (6). Although the incidence has remained low for decades, a resurgence has been observed since the late 1990s, including outbreaks in high-income countries (7). In the United States, 48,277 cases were reported in 2012, corresponding to an incidence of more than 10 cases per 100,000, with the highest in 50 years. Similarly, Australia recorded an incidence of 47.7 per 100,000 in 2019 (8). According to the WHO estimates, 24.1 million pertussis cases occur annually among children under five, resulting in approximately 160,700 deaths worldwide (9).

In Saudi Arabia, as in other parts of the world, pertussis continues to pose challenges, despite the establishment of immunization programs. Global resurgence has been particularly evident since the COVID-19 pandemic, with increasing numbers of cases documented in Europe and China (10). This resurgence has been attributed in part to the relaxation of pandemic-related restrictions, resulting in an “immunity debt” due to reduced exposure to pathogens (11).

The present study investigated the epidemiology of pertussis in Saudi Arabia from 2006 to 2023 by examining the incidence across age groups, regions, sexes, and nationalities. It further explored the influence of vaccination strategies and public health measures, particularly in the context of recent global trends and evolving *Bordetella pertussis* strains. By analyzing nearly two decades of national data, this study aimed to enhance our understanding of pertussis epidemiology and inform public health interventions.

To the best of our knowledge, this is the first comprehensive study to evaluate the epidemiological patterns of pertussis across all regions of Saudi Arabia. This study provides critical insights into the regional variations, age-related susceptibility, and impact of public health measures, particularly during and after the COVID-19 pandemic. Ultimately, these findings may guide future vaccination strategies and strengthen national responses to pertussis outbreaks.

## 2 Methods

### 2.1 Study setting and population

This retrospective descriptive study included all the recorded cases of pertussis in Saudi Arabia between January 2006 and December 2023. The analysis encompassed the entire Saudi population stratified by demographic and regional characteristics.

### 2.2 Study design and duration

A retrospective design was employed to analyze national epidemiological data over an 18-year period (2006–2023). This study examined the temporal and demographic variations in the incidence of pertussis across multiple cohorts.

### 2.3 Data collection

Data were obtained from the Statistical Yearbooks of the Saudi Ministry of Health, which is a comprehensive repository of national health statistics. All officially reported pertussis cases during the study period were included. Ethical approval was not required because the data were publicly available. The dataset covers 20 administrative regions in Saudi Arabia and provides information on both case numbers and population denominators. The crude incidence rate (CIR) was calculated annually as the number of newly reported cases per 100,000 individuals.

### 2.4 Study measurements

The variables extracted included the annual number of cases and CIR, categorized by year of diagnosis, age group, region, sex, and nationality (Saudi vs. non-Saudi).

### 2.5 Data management and analysis

All statistical analyses were performed using SPSS (version 20.0; IBM Corp., Armonk, NY, USA). Descriptive statistics were applied to summarize the total number of pertussis cases and corresponding crude incidence rates (CIRs) per 100,000 people. The results were stratified by year of diagnosis, age group (0–4 years, 5–14 years, 15–44 years, and ≥45 years), geographic region, sex, and nationality (Saudi vs. non-Saudi). In addition, age-specific incidence rates (ASIRs) were calculated for 2023 to provide more detailed insights into the distribution of disease burden across the population.

The geographic distribution of pertussis was analyzed by comparing the CIRs across 20 administrative regions of Saudi Arabia as reported in the Ministry of Health Statistical Yearbooks between 2006 and 2023. An inferential statistical analysis was performed. The Shapiro–Wilk test was applied to assess the normality of the CIR data across study variables, with a *p*-value greater than 0.05, which was considered evidence of a normal distribution. To compare the mean CIRs between Saudi and non-Saudi populations, an independent-samples *t*-test was employed when the assumption of normality was satisfied. Levene’s test indicated unequal variances, and the adjusted t-test results are reported. For comparisons across multiple groups, including different years of diagnosis, age categories, and regions, the Kruskal–Wallis H test was used as a non-parametric alternative to one-way ANOVA, given that most subgroup data were not normally distributed. The chi-square approximation was applied to evaluate the overall group differences, and the results were reported with corresponding test statistics and *p*-values.

## 3 Results

### 3.1 Annual trend of pertussis cases by year of diagnosis

**Figure 1:**
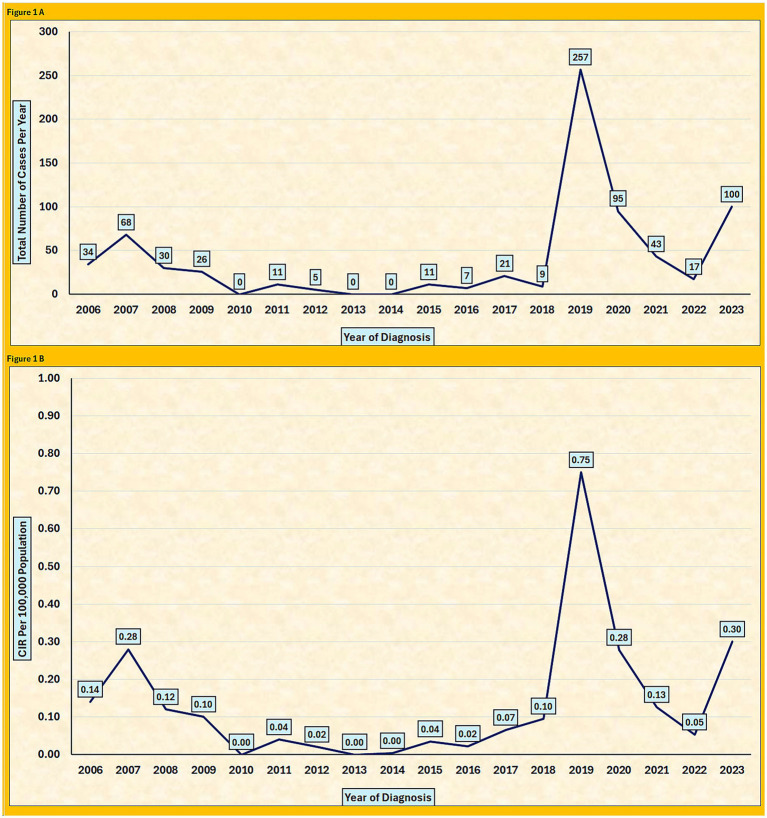
**(A)** Annual number of reported pertussis cases in Saudi Arabia, 2006–2023. **(B)** Yearly crude incidence rate (CIR) of pertussis per 100,000 population in Saudi Arabia, 2006–2023.

From 2006 to 2023, the annual number of reported cases of pertussis in Saudi Arabia will fluctuate considerably. A peak was observed in 2007, with 68 cases, followed by a marked decline between 2010 and 2017, with the incidence reaching zero in 2010, 2013, and 2014. A substantial outbreak occurred in 2019 when the number of cases increased sharply to 257. This was followed by a decline to 95 cases in 2020 and a further decrease to 43 and 17 cases in 2021 and 2022, respectively. In 2023, however, the number of cases will increase to 100 (Figure 1A).

The crude incidence rate (CIR) per 100,000 individuals showed a similar pattern. The CIR was 0.14 in 2006, the CIR rose to 0.28 in 2007, after which it declined steadily between 2008 and 2018, reaching 0.00 in years with no reported cases (2010, 2013, and 2014). In 2019, the CIR peaked at 0.75, the highest during the entire study period, before decreasing to 0.28 2020, 0.13 2021, and 0.05 in 2022. A rebound is observed in 2023, with the CIR increasing to 0.30 (Figure 1B).

### 3.2 Distribution of pertussis cases by age group

Between 2006 and 2023, infants under 1 year of age accounted for the majority of pertussis cases, with a total of 610 cases and a mean of 34 cases per year. This age group had the highest disease burden across all age categories. Children aged 1–4 years followed 63 cases (mean, 4 cases per year), while older children (5–14 years) followed 23 cases in total (mean, 1 case per year). Adults aged 15–44 years and those aged ≥45 years contributed only 22 and 17 cases, respectively, over the entire study period (Table 1).

**Table 1:** Total number and distribution of pertussis cases by age group in Saudi Arabia, 2006–2023, & age-specific crude incidence rate (CIR) of pertussis per 100,000 population in Saudi Arabia, 2023.

Year of diagnosis	<1	(1–4)	(5–14)	(15–44)	≥45	Total
2006	24	6	4	0	0	34
2007	42	13	5	5	3	68
2008	26	2	0	0	2	30
2009	24	1	0	1	0	26
2010	0	0	0	0	0	0
2011	11	0	0	0	0	11
2012	5	0	0	0	0	5
2013	0	0	0	0	0	0
2014	1	0	0	0	0	1
2015	11	0	0	0	0	11
2016	6	1	0	0	0	7
2017	14	0	1	5	1	21
2018	7	1	1	0	0	9
2019	236	8	5	4	4	257
2020	81	8	2	2	2	95
2021	37	3	0	1	2	43
2022	15	2	0	0	0	17
2023	70	18	5	4	3	100
Total	610	63	23	22	17	735
Mean	34	4	1	1	1	41
Median	15	1	0	0	0	

Age group	Total population 2023	Number of cases	CIR
0 to 4	2,578,159	88	3.4
5 to 14	5,308,731	5	0.09
15 to 44	18,570,062	4	0.02
≥45	5,718,272	3	0.05
All ages	32,175,224	100	0.03

Tests of normality (Kolmogorov–Smirnov and Shapiro–Wilk) confirmed that the data for all age groups were not normally distributed (*p* < 0.05). Accordingly, the Kruskal–Wallis test was applied and revealed significant variation in pertussis distribution across the five age categories [*χ*^2^(4, *N* = 735) = 30.464, *p* < 0.001]. The highest median case count was observed in infants (<1 year; median 15 cases), whereas all older age groups demonstrated substantially lower medians (1 case in the 1–4 years group and 0 cases in the 5–14, 15–44, and ≥45 years groups).

The Age-Specific Incidence Rate (ASIR) of pertussis in 2023 was calculated for the different age groups to understand the distribution of cases across populations. For the age group of 0–4 years, the CIR was 3.4 cases per 100,000 population, with 88 cases reported among a population of 2,578,159 individuals. In the 5–14 year age group, the CIR was 0.09 cases per 100,000 population, based on five reported cases out of a total population of 5,308,731. Similarly, in the 15 to 44 years age group, the CIR was 0.02 cases per 100,000 population, with four reported cases among 18,570,062 individuals. The oldest age group, 45 years and above, had a CIR of 0.05 cases per 100,000 based on three cases reported in a population of 5,718,272. These findings indicate that the highest incidence of pertussis occurred in children under 4 years of age, while significantly lower incidence rates were observed in older age groups. The overall ASIR for the entire population was 0.3 cases per 100,000, based on 100 total cases from a population of 32,175,224 (Table 1).

### 3.3 Distribution of pertussis cases by region

Based on the data from the Ministry of Health Statistical Yearbooks, regional variations in pertussis cases were observed across Saudi Arabia between 2006 and 2023. Riyadh reported the highest total number of cases, with 143 recorded over the study period, corresponding to an average of eight cases per year. Aseer followed up 98 cases (mean, five cases per year), while Jeddah reported a total of 97 cases (mean, five cases per year). Similarly, the Eastern Region accounted for 94 cases (mean, five cases per year), and Al-Ahsa reported 53 cases (mean, three cases per year).

Moderate numbers have also been documented in other regions. Qaseem reported 77 cases (mean, 4 cases per year), Taif recorded 66 cases (mean, 4 cases per year), and Hafr Al-Baten reported 13 cases (mean, 1 case per year). Medinah reported 28 cases (mean, two cases per year), whereas Makkah documented 19 cases (mean, one case per year). Lower totals were also noted in Jazan (23 cases), Najran (three cases), Al-Bahah (two cases), Bishah (two cases), Tabouk (four cases), Hail (eight cases), and Qunfudah (four cases). By contrast, several regions, including Al-Jouf and Qurayyat, reported no cases of pertussis throughout the 18-year study period. Overall, across all 20 administrative regions, 735 pertussis cases were reported between 2006 and 2023, with an average of 41 cases per year (Table 2).

**Table 2:** Total number of reported pertussis cases by region in Saudi Arabia, 2006–2023.

Region	2006	2007	2008	2009	2010	2011	2012	2013	2014	2015	2016	2017	2018	2019	2020	2021	2022	2023	Total	Overall Per Year
Riyadh	3	1	0	0	0	9	2	0	0	7	0	3	1	55	14	8	5	35	143	8
Makkah	1	8	5	0	0	0	0	0	0	0	1	0	1	2	1	0	0	0	19	1
Jeddah	8	3	7	0	0	0	0	0	0	0	2	7	1	28	17	5	2	17	97	5
Taif	0	3	0	0	0	0	0	0	0	0	0	0	1	38	13	6	0	5	66	4
Medinah	0	0	0	25	0	0	0	0	0	0	0	0	0	1	2	0	0	0	28	2
Qaseem	16	20	14	1	0	1	2	0	1	0	1	0	0	12	4	0	0	5	77	4
Eastern	1	18	1	0	0	1	1	0	0	0	2	6	1	30	9	8	6	10	94	5
Al-Ahsa	0	10	1	0	0	0	0	0	0	2	1	2	0	23	4	1	1	8	53	3
Hafr Al-Baten	0	0	0	0	0	0	0	0	0	0	0	0	1	6	1	1	0	4	13	1
Aseer	2	2	1	0	0	0	0	0	0	1	0	0	1	44	25	11	3	8	98	5
Bishah	0	0	1	0	0	0	0	0	0	0	0	0	0	0	0	0	0	1	2	0
Tabouk	0	0	0	0	0	0	0	0	0	0	0	3	0	0	1	0	0	0	4	0
Hail	0	0	0	0	0	0	0	0	0	0	0	0	1	5	0	0	0	2	8	0
Northern	0	0	0	0	0	0	0	0	0	0	0	0	0	1	0	0	0	0	1	0
Jazan	0	3	0	0	0	0	0	0	0	1	0	0	1	10	4	2	0	2	23	1
Najran	0	0	0	0	0	0	0	0	0	0	0	0	0	0	0	0	0	3	3	0
Al-Bahah	0	0	0	0	0	0	0	0	0	0	0	0	0	2	0	0	0	0	2	0
Al-Jouf	0	0	0	0	0	0	0	0	0	0	0	0	0	0	0	0	0	0	0	0
Qurayyat	0	0	0	0	0	0	0	0	0	0	0	0	0	0	0	0	0	0	0	0
Qunfudah	3	0	0	0	0	0	0	0	0	0	0	0	0	0	0	1	0	0	4	0
Total	34	68	30	26	0	11	5	0	1	11	7	21	9	257	95	43	17	100	735	41

**Figure 2:**
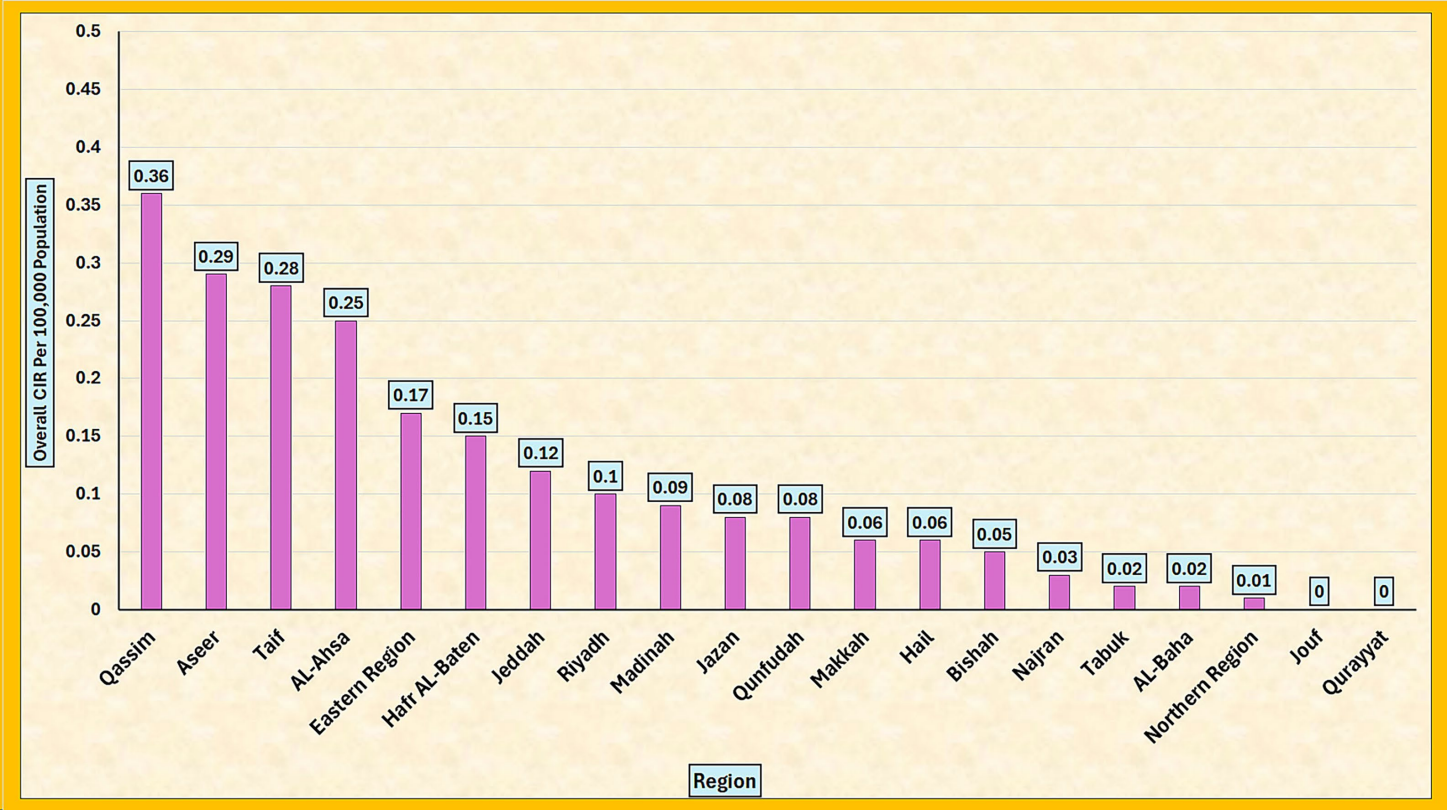
Overall crude incidence rate (CIR) of pertussis per 100,000 population by region in Saudi Arabia, 2006–2023.

The overall crude incidence rate (CIR) of pertussis per 100,000 people varied markedly across the regions of Saudi Arabia during the study period, as presented in Table 3 and illustrated in Figure 2. The highest overall CIRs were recorded for Qassim (0.36), Aseer (0.29), Taif (0.28), and Al-Ahsa (0.25). These four regions consistently showed higher pertussis incidence than the rest of the country. In contrast, lower CIRs were documented in several regions, including Najran (0.03), Tabuk (0.02), Al-Baha (0.02), and the Northern Region (0.01). Notably, no cases were reported by Jouf and Qurayyat throughout the study period, resulting in an overall CIR of 0.00 in these regions. Riyadh, Jeddah, and the Eastern Region had intermediate CIRs of 0.10, 0.12, and 0.17. The Kruskal–Wallis test confirmed that the observed differences between regions were statistically significant [*χ*^2^(19, *N* = 735) = 87.835, *p* < 0.001], demonstrating substantial geographic variation in pertussis incidence across the Kingdom.

**Table 3:** Crude incidence rate (CIR) of pertussis per 100,000 population by region in Saudi Arabia, 2006–2023.

Region	Overall CIR of Pertussis in Saudi Arabia Per 100,000 from 2006 to 2023																		Overall CIR
	2006	2007	2008	2009	2010	2011	2012	2013	2014	2015	2016	2017	2018	2019	2020	2021	2022	2023	
Riyadh	0.05	0.02	0.00	0.00	0.00	0.13	0.03	0.00	0.00	0.09	0.00	0.04	0.01	0.64	0.16	0.09	0.06	0.41	0.10
Makkah	0.07	0.51	0.31	0.00	0.00	0.00	0.00	0.00	0.00	0.00	0.04	0.00	0.04	0.08	0.04	0.00	0.00	0.00	0.06
Jeddah	0.24	0.09	0.21	0.00	0.00	0.00	0.00	0.00	0.00	0.00	0.04	0.15	0.02	0.57	0.34	0.10	0.05	0.43	0.12
Taif	0.00	0.28	0.00	0.00	0.00	0.00	0.00	0.00	0.00	0.00	0.00	0.00	0.08	2.79	0.94	0.43	0.00	0.45	0.28
Madinah	0.00	0.00	0.00	1.45	0.00	0.00	0.00	0.00	0.00	0.00	0.00	0.00	0.00	0.04	0.09	0.00	0.00	0.00	0.09
Qassim	1.50	1.84	1.26	0.09	0.00	0.08	0.15	0.00	0.07	0.00	0.07	0.00	0.00	0.81	0.26	0.00	0.00	0.37	0.36
Eastern Region	0.05	0.80	0.04	0.00	0.00	0.04	0.03	0.00	0.00	0.00	0.06	0.19	0.03	0.88	0.26	0.23	0.17	0.28	0.17
AL-Ahsa	0.00	1.03	0.10	0.00	0.00	0.00	0.00	0.00	0.00	0.16	0.08	0.16	0.00	1.80	0.31	0.08	0.09	0.72	0.25
Hafr AL-Baten	0.00	0.00	0.00	0.00	0.00	0.00	0.00	0.00	0.00	0.00	0.00	0.00	0.22	1.28	0.21	0.21	0.00	0.86	0.15
Aseer	0.13	0.13	0.06	0.00	0.00	0.00	0.00	0.00	0.00	0.06	0.00	0.00	0.05	2.31	1.29	0.57	0.17	0.47	0.29
Bishah	0.00	0.00	0.49	0.00	0.00	0.00	0.00	0.00	0.00	0.00	0.00	0.00	0.00	0.00	0.00	0.00	0.00	0.32	0.05
Tabuk	0.00	0.00	0.00	0.00	0.00	0.00	0.00	0.00	0.00	0.00	0.00	0.33	0.00	0.00	0.10	0.00	0.00	0.00	0.02
Hail	0.00	0.00	0.00	0.00	0.00	0.00	0.00	0.00	0.00	0.00	0.00	0.00	0.14	0.68	0.00	0.00	0.00	0.27	0.06
Northern Region	0.00	0.00	0.00	0.00	0.00	0.00	0.00	0.00	0.00	0.00	0.00	0.00	0.00	0.26	0.00	0.00	0.00	0.00	0.01
Jazan	0.00	0.23	0.00	0.00	0.00	0.00	0.00	0.00	0.00	0.06	0.00	0.00	0.06	0.61	0.24	0.12	0.00	0.14	0.08
Najran	0.00	0.00	0.00	0.00	0.00	0.00	0.00	0.00	0.00	0.00	0.00	0.00	0.00	0.00	0.00	0.00	0.00	0.51	0.03
AL-Baha	0.00	0.00	0.00	0.00	0.00	0.00	0.00	0.00	0.00	0.00	0.00	0.00	0.00	0.40	0.00	0.00	0.00	0.00	0.02
Jouf	0.00	0.00	0.00	0.00	0.00	0.00	0.00	0.00	0.00	0.00	0.00	0.00	0.00	0.00	0.00	0.00	0.00	0.00	0.00
Qurayyat	0.00	0.00	0.00	0.00	0.00	0.00	0.00	0.00	0.00	0.00	0.00	0.00	0.00	0.00	0.00	0.00	0.00	0.00	0.00
Qunfudah	1.19	0.00	0.00	0.00	0.00	0.00	0.00	0.00	0.00	0.00	0.00	0.00	0.00	0.00	0.00	0.30	0.00	0.00	0.08

### 3.4 Distribution of pertussis cases by nationality

#### 3.4.1 Saudi nationals

**Figure 3:**
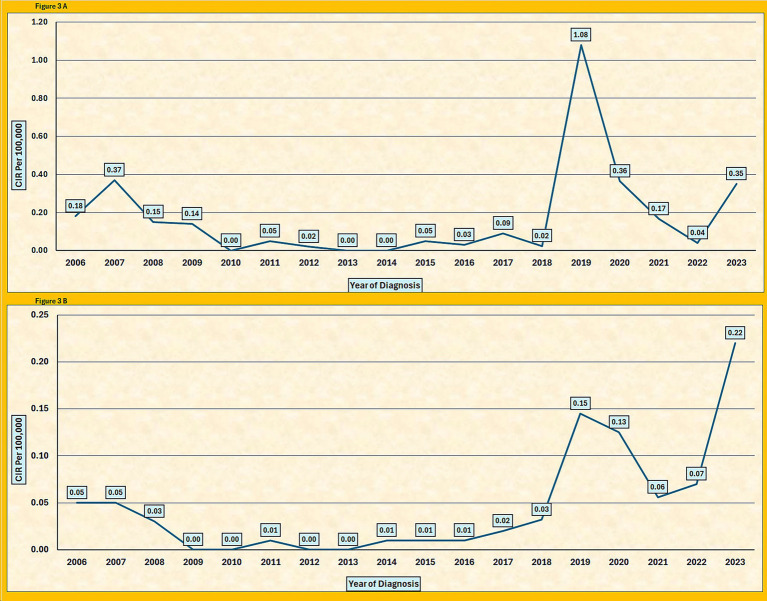
**(A)** Overall crude incidence rate (CIR) of pertussis per 100,000 population among Saudi nationals, 2006–2023. **(B)** Overall crude incidence rate (CIR) of pertussis per 100,000 population among non-Saudi nationals, 2006–2023.

Between 2006 and 2023, 628 pertussis cases were recorded among Saudi nationals, with an average of 35 cases per year. Of these, 321 (51%) were male and 312 (49%) were female, with a male-to-female ratio of 1.1:1. The CIR for Saudi nationals varied over the years, starting at 0.18 per 100,000 people in 2006 and peaking at 1.08 per 100,000 in 2019. Following this peak, the CIR declines, reaching 0.04 per 100,000 in 2022, before slightly increasing again to 0.35 per 100,000 in 2023. The CIR across the years exhibited notable fluctuations, with significant increases observed in 2019 and 2023 (Table 4 and Figure 3A).

**Table 4:** Crude incidence rate (CIR) of pertussis per 100,000 population by nationality in Saudi Arabia, 2006–2023.

CIR of Pertussis cases by nationality											
Year of diagnosis	Saudi				Ratio cases	Ratio CIR	Non-Saudi				Ratio cases
	Male	Female	Total	CIR	Male Saudi / Female Saudi	Saudi / Non-Saudi	Male	Female	Total	CIR	Male Non-Saudi / Female Non Saudi
2006	20	11	31	0.18	1.8	3.6	2	1	3	0.05	2.0
2007	41	24	65	0.37	1.7	7.4	2	1	3	0.05	2.0
2008	16	12	28	0.15	1.3	5.0	1	1	2	0.03	1.0
2009	11	15	26	0.14	0.7	N/A	0	0	0	0.00	0.0
2010	0	0	0	0.00	0.0	N/A	0	0	0	0.00	0.0
2011	5	5	10	0.05	1.0	5.0	0	1	1	0.01	0.0
2012	2	3	0	0.02	0.7	N/A	0	0	0	0.00	0.0
2013	0	0	0	0.00	0.0	N/A	0	0	0	0.00	0.0
2014	0	0	0	0.00	0.0	0.0	1	0	1	0.01	0.0
2015	4	6	10	0.05	0.7	5.0	1	0	1	0.01	0.0
2016	4	2	6	0.03	2.0	3.0	1	0	1	0.01	0.0
2017	8	10	18	0.09	0.8	4.5	1	2	3	0.02	0.5
2018	2	3	5	0.02	0.7	0.8	2	2	4	0.03	1.0
2019	104	134	238	1.08	0.8	7.4	8	11	19	0.15	0.7
2020	47	31	78	0.36	1.5	2.9	8	9	17	0.13	0.9
2021	14	22	36	0.17	0.6	3.0	5	2	7	0.06	2.5
2022	6	1	7	0.04	6.0	0.6	4	6	10	0.07	0.7
2023	37	33	70	0.35	1.1	1.6	12	18	30	0.22	0.7
Total	321	312	628	N/A	1.0	N/A	48	54	102	0.05	0.9
Overall	18	17	35	0.17	1.1	3.6	3	3	6	0.05	1.0

#### 3.4.2 Non-Saudi nationals

For non-Saudi nationals, 102 pertussis cases were recorded over the same period, with an average of six cases per year. Of these patients, 48 (47%) were male and 54 (53%) were female, with a male-to-female ratio of 0.9:1. The CIR for non-Saudis remains consistently lower than that for Saudi nationals, peaking at 0.15 per 100,000 in 2019 and gradually decreasing to 0.22 per 100,000 by 2023 (Figure 3B).

An independent samples *t*-test was conducted to examine the differences in the Crude Incidence Rate (CIR) of pertussis between the Saudi and non-Saudi populations. The Levene’s test for the equality of variances indicated that the assumption of equal variances was violated (*F* = 6.439, *p* = 0.016), indicating that the variances between the two groups were significantly different. Consequently, t-test results without assuming equal variances were considered. The t-test for equality of means showed that there was no statistically significant difference in the CIR between the Saudi and non-Saudi populations [t(18.894) = 1.992, *p* = 0.061]. The mean difference in the CIR between the groups was 0.125, with a 95% confidence interval (CI) ranging from −0.0064 to 0.2564, suggesting that although the Saudi population had a slightly higher CIR, the difference was not statistically significant.

## 4 Discussion

Pertussis continues to pose a major public health challenge in Saudi Arabia and requires sustained epidemiological monitoring and targeted control strategies. The current study provides one of the most comprehensive analyses of the incidence of pertussis in the Kingdom, covering nearly two decades of surveillance data. Characterizing temporal, demographic, and regional variations offers critical insights that can support vaccination strategies and strengthen public health responses.

The epidemiological profile of pertussis from 2006 to 2023 revealed notable fluctuations in incidence. The relatively stable case numbers during the early years, followed by a peak in 2007, may reflect the variability in vaccine coverage or reporting accuracy. The subsequent decline, with no cases reported in 2010, 2013, or 2014, could be related to underreporting, temporary improvements in herd immunity, or other epidemiological factors.

**Figure 4:**
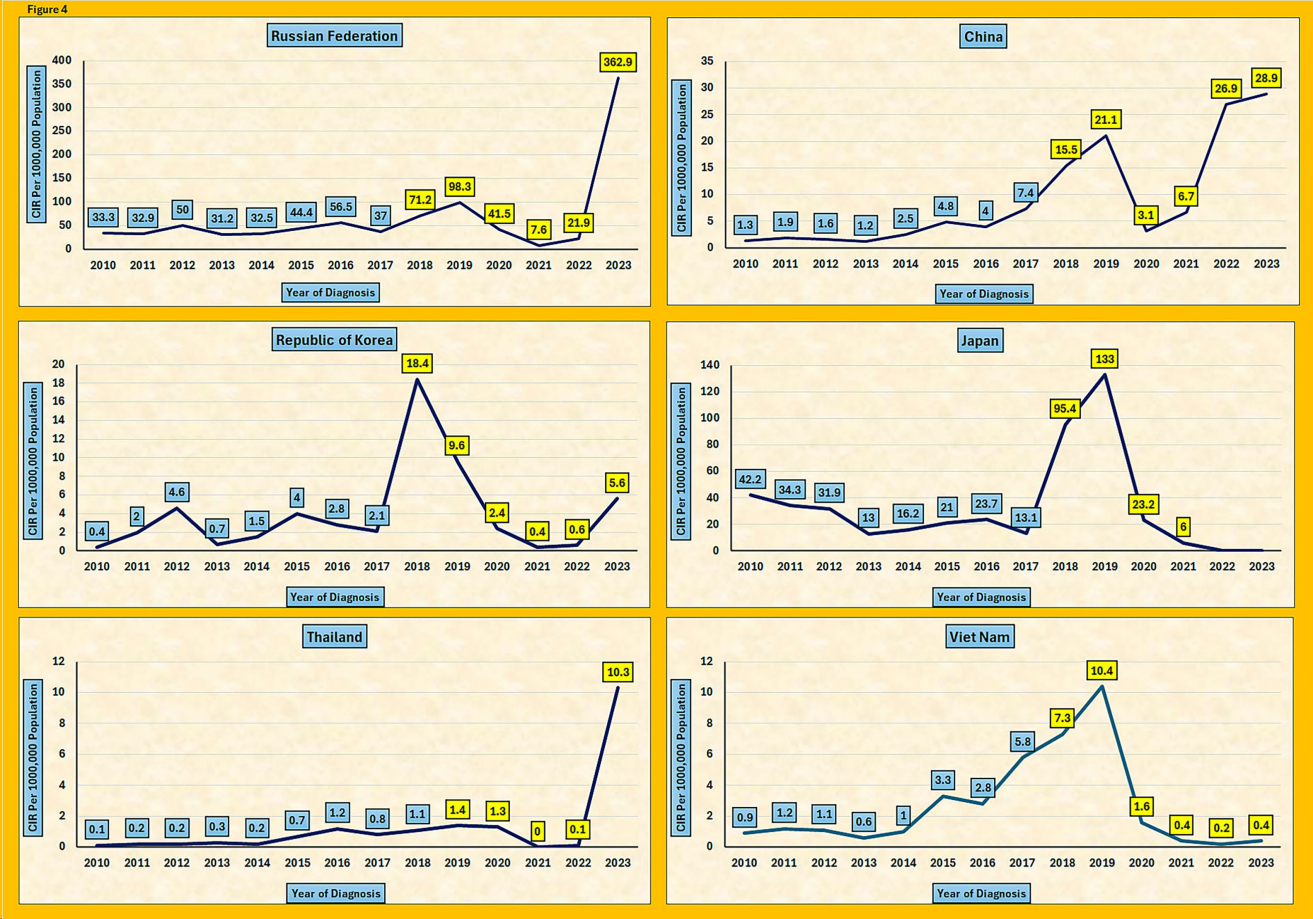
Trends in pertussis crude incidence rates (CIR) across six countries, 2010–2023.

A pronounced surge in 2019, with 257 reported cases, marked a major outbreak in Saudi Arabia. Similar increases were reported worldwide. For example, in the Russian Federation, the crude incidence rate (CIR) rose from 98.3 per 1,000,000 in 2019 to 362.9 in 2023, whereas in Japan, the rate increased from 95.4 in 2018 to 133 per 1,000,000 in 2019. Comparable spikes were also observed in China, Republic of Korea, Thailand, and Vietnam, reflecting a global pattern of pertussis resurgence (Figure 4) (13). The synchronous rise across multiple countries points to shared factors, such as waning immunity from the acellular vaccine, gaps in vaccination coverage, and improved diagnostic capabilities. The decline in cases after 2019 in Saudi Arabia may reflect strengthened public health measures; however, the resurgence in 2023, seen nationally and internationally, highlights the persistent challenges in pertussis control.

Age-specific analysis confirmed that infants younger than 1 year were the most affected group in Saudi Arabia, with 610 cases reported over the study period (mean, 34 cases annually). This finding is consistent with the global data. The CDC regularly reports that infants under 12 months account for the highest pertussis incidence worldwide (14). Similarly, a study from Tianjin, China, demonstrated that younger age and incomplete vaccination schedules significantly increased the risk of severe pertussis (15). These findings emphasize the well-documented vulnerability of infants compared to older children and adults.

The geographic distribution of the cases also demonstrated marked variation. Regions such as Qassim (CIR 0.36), Aseer (0.29), Taif (0.28), and Al-Ahsa (0.25) reported the highest incidences, whereas Najran (0.03), Tabuk (0.02), Al-Baha (0.02), and the Northern Region (0.01) reported the lowest incidences. Jouf et al. and Qurayyat et al. did not record any cases during their study period. These differences may be linked to variations in the vaccination coverage, timeliness of booster doses, and healthcare access. A previous national study documented regional disparities in healthcare access between the urban and rural areas of Saudi Arabia, which may partially explain these differences (16). The Kruskal–Wallis test confirmed the statistical significance of regional disparities in CIR, further highlighting the role of regional healthcare systems in shaping pertussis epidemiology (12).

The analysis by nationality revealed further distinctions. Saudi nationals accounted for 628 cases (86%), whereas non-Saudis reported 102 cases during the study period. The CIR among Saudis peaked in 2019 at 1.08 per 100,000, compared to 0.15 per 100,000 among non-Saudis. The male-to-female ratio was nearly equal in both groups (1.1:1 among Saudis; 0.9:1 among non-Saudis). Although cases were more frequent among Saudis, statistical testing showed no significant difference in the incidence rates between Saudis and non-Saudis (*p* = 0.061). These findings suggest that factors such as age, regional healthcare access, and population structure may play a greater role than nationality in shaping the risk of pertussis.

## 5 Study limitations

This study has several limitations. First, it relied on secondary data obtained from national surveillance reports, which may be subject to underreporting, misclassification, or inconsistent diagnostic practices across regions and over time. These limitations could affect the accuracy and completeness of the reported incidence rates of pertussis. Second, the dataset lacked information on individual-level vaccination status, making it impossible to assess the relationship among vaccination coverage, schedule adherence, and pertussis trends. Third, the analysis did not incorporate potential confounding variables such as socioeconomic status, urban–rural residence, or healthcare accessibility, which may influence both the likelihood of infection and case detection. Fourth, the study period included the COVID-19 pandemic years (2020–2022), during which changes in healthcare-seeking behavior, diagnostic focus, and public health interventions (e.g., lockdown and mask use) likely altered the transmission dynamics of pertussis. Finally, the retrospective and descriptive nature of the study limits causal inferences. Future studies should aim to incorporate prospective data collection, individual-level immunization histories, and broader contextual variables to better understand the epidemiology of pertussis and guide more tailored public health responses.

## 6 Conclusion

This study offers key insights into the epidemiology of pertussis in Saudi Arabia from 2006 to 2023, revealing significant variations in the incidence by age, region, and nationality. Infants under 1 year of age had the highest burden, underscoring the need for targeted vaccination. Regional disparities with a higher incidence in Qassim, Aseer, and Taif highlight the need for region-specific public health interventions. These fluctuating trends, including a peak in 2019 and a resurgence in 2023, emphasize the importance of ongoing vigilance and vaccination strategies to control future outbreaks.

## Data Availability

Publicly available datasets were analyzed in this study. This data can be found here: https://www.moh.gov.sa/en/Ministry/Statistics/book/Pages/default.aspx.
